# Secondary polycythemia in chronic obstructive pulmonary disease: prevalence and risk factors

**DOI:** 10.1186/s12890-021-01585-5

**Published:** 2021-07-14

**Authors:** Jingzhou Zhang, Dawn L. DeMeo, Edwin K. Silverman, Barry J. Make, R. Chad Wade, J. Michael Wells, Michael H. Cho, Brian D. Hobbs

**Affiliations:** 1 Department of Medicine, Mount Auburn Hospital, Harvard Medical School, Cambridge, MA USA; 2 Channing Division of Network Medicine, Brigham and Women’s Hospital, Harvard Medical School, Boston, MA USA; 3 Division of Pulmonary and Critical Care Medicine, Brigham and Women’s Hospital, Harvard Medical School, Boston, MA USA; 4 Division of Pulmonary, Critical Care and Sleep Medicine, National Jewish Health, Denver, CO USA; 5 Lung Health Center and the Division of Pulmonary, Allergy, and Critical Care Medicine, University of Alabama at Birmingham (UAB), Birmingham, AL USA; 6 Birmingham VA Medical Center, Birmingham, AL USA

**Keywords:** COPD, DLCO, Hypoxemia, Oxygen therapy, Polycythemia

## Abstract

**Background:**

Secondary polycythemia is associated with cigarette smoking and chronic obstructive pulmonary disease (COPD). However, the prevalence of polycythemia in COPD and the contributing risk factors for polycythemia in COPD have not been extensively studied.

**Methods:**

We analyzed the presence of secondary polycythemia in current and former smokers with moderate to very severe COPD at the five-year follow-up visit in the observational COPDGene study. We used logistic regression to evaluate the association of polycythemia with age, sex, race, altitude, current smoking status, spirometry, diffusing capacity for carbon monoxide (DLCO), quantitative chest CT measurements (including emphysema, airway wall thickness, and pulmonary artery to aorta diameter ratio), resting hypoxemia, exercise-induced hypoxemia, and long-term oxygen therapy.

**Results:**

In a total of 1928 COPDGene participants with moderate to very severe COPD, secondary polycythemia was found in 97 (9.2%) male and 31 (3.5%) female participants. In a multivariable logistic model, severe resting hypoxemia (OR 3.50, 95% CI 1.41–8.66), impaired DLCO (OR 1.28 for each 10-percent decrease in DLCO % predicted, CI 1.09–1.49), male sex (OR 3.60, CI 2.20–5.90), non-Hispanic white race (OR 3.33, CI 1.71–6.50), current smoking (OR 2.55, CI 1.49–4.38), and enrollment in the Denver clinical center (OR 4.42, CI 2.38–8.21) were associated with higher risk for polycythemia. In addition, continuous (OR 0.13, CI 0.05–0.35) and nocturnal (OR 0.46, CI 0.21–0.97) supplemental oxygen were associated with lower risk for polycythemia. Results were similar after excluding participants with anemia and participants enrolled at the Denver clinical center.

**Conclusions:**

In a large cohort of individuals with moderate to very severe COPD, male sex, current smoking, enrollment at the Denver clinical center, impaired DLCO, and severe hypoxemia were associated with increased risk for secondary polycythemia. Continuous or nocturnal supplemental oxygen use were associated with decreased risk for polycythemia.

**Supplementary Information:**

The online version contains supplementary material available at 10.1186/s12890-021-01585-5.

## Background

Cigarette smoking and chronic obstructive pulmonary disease (COPD) are associated with secondary polycythemia [1, 2], which may contribute to pulmonary hypertension (PH) [3, 4], venous thromboembolism [5–7], and mortality in COPD [8–11].

Polycythemia prevalence in COPD outpatients ranges from 6 to 10.2% when defined by a hemoglobin ≥ 17 g/dL in males and ≥ 15 g/dL in females [9, 12, 13]. The prevalence of a hematocrit ≥ 55% was 8.4% in a sample of patients with severe COPD receiving long-term oxygen therapy (LTOT) [11]. Polycythemia in COPD has been noted to be less frequent following the widespread use of LTOT [14], yet a secular trend in polycythemia prevalence has not been well-established due to a lack of comparable samples of COPD patients across the full spectrum of disease severity. Additionally, in 2016 the World Health Organization (WHO) redefined polycythemia vera (a myeloproliferative neoplasm) as a hemoglobin > 16.5 g/dL and/or a hematocrit > 49% in males and a hemoglobin > 16.0 g/dL and/or a hematocrit > 48% in females [15]. The diagnosis of secondary polycythemia is extrapolated from the WHO definition for polycythemia vera. The prevalence of secondary polycythemia is unclear in a contemporary COPD population due to the recent change in the definition of polycythemia and an increased use of LTOT in COPD patients.

Increased carboxyhemoglobin (COHb) in active smokers and chronic hypoxemia in COPD patients are proposed to contribute to the development of secondary polycythemia; however, the underlying physiological mechanisms are not fully understood [1, 14, 16]. Hematocrit has been negatively correlated with: age, forced expiratory volume in 1 s (FEV_1_) percent predicted, and ratio of FEV_1_ to forced vital capacity (FVC); and positively correlated with: male sex, current smoking, partial pressure of carbon dioxide in the blood, and body mass index (BMI) in COPD patients [11]. However, secondary polycythemia risk factors have not been examined in an extensively phenotyped sample of COPD patients with and without hypoxemia. This study aims to evaluate the prevalence and risk factors of secondary polycythemia in a cohort of current- and former-smoking COPD patients in the Genetic Epidemiology of COPD (COPDGene) study. Part of the results of this study were published as an abstract for the 2020 American Thoracic Society annual meeting.

## Methods

### Study design and population

We performed a cross-sectional analysis of selected participants from the COPDGene study (ClinicalTrials.gov Identifier: NCT00608764), an ongoing prospective observational study involving twenty-one clinical centers across the United States. Study design and methodology for COPDGene have been previously reported and are available online at www.COPDGene.org [17]. Briefly, the COPDGene study included participants aged 45 to 80 years with self-identified race/ethnicity of non-Hispanic white or African American and at least a 10 pack-year smoking history. Participants of the COPDGene study were asked to complete a demographic questionnaire and report their self-identified race as White, Black or African American, Asian, Pacific Islander, American Indian, and Other. The participants were also asked to report their self-identified ethnicity as Hispanic or Latino vs. Not Hispanic or Latino. Only participants identifying as “White” or “Black/African American” and “Not Hispanic or Latino” were eligible to participate given the study design of COPDGene study. Ancestry was assessed using genetic data and was confirmed to match well with self-identified race [18]. Current smoking status and pack-years of cigarette smoking were obtained based on self-report. COPD-related phenotypes including clinical, physiological, laboratory, and imaging features were collected in COPDGene. Our polycythemia investigation included participants from the COPDGene 5-year-follow-up visit with Global Initiative for Chronic Obstructive Lung Disease (GOLD) grade 2–4 airflow limitation severity (post-bronchodilator FEV_1_/FVC < 0.7 and FEV_1_% predicted < 80%) and available complete blood count (CBC) data. All phenotypic data were from the COPDGene 5-year follow-up visit with the exception of main pulmonary artery to ascending aorta diameter ratio (PA/A) which was obtained at the baseline COPDGene visit. The COPDGene study was approved by the respective Institutional Review Boards at all participating clinical centers, which are outlined in the Additional file 2. Written informed consent was obtained from each study participant.

### Polycythemia

The assignment of a secondary polycythemia diagnosis was extrapolated from the 2016 WHO diagnostic criteria for the myeloproliferative neoplasm, polycythemia vera (PV): hemoglobin > 16.5 g/dL and/or hematocrit > 49% in males; hemoglobin > 16.0 g/dL and/or hematocrit > 48% in females [15]. The hemoglobin and hematocrit values were obtained from CBC data obtained at the COPDGene 5-year follow-up visit.

### Pulmonary function testing

Spirometry and the diffusing capacity of the lungs for carbon monoxide (DLCO) were measured using the ndd EasyOne system (Zurich, Switzerland) in accordance with the 2005 ATS/ERS guidelines [19]. Spirometry was measured before and after administration of inhaled albuterol, and postbronchodilator spirometry was used in the present study. Extensive quality control of pulmonary function test data was performed by both an automated system and manual review. FEV_1_ and FVC percent predicted values were calculated with the National Health and Nutrition Examination Survey III equations [20]. DLCO was adjusted for hemoglobin and altitude, and DLCO percent predicted values were calculated based on the Global Lung Initiative 2012 reference equations [21, 22].

### Chest computed tomography imaging

All participants had inspiratory and expiratory chest CT scans using whole-lung volumetric multidetector CT scanners with a standardized protocol [17]. Quantitative analysis of CT scans was performed using LungQ software (Thirona, Nijmegen, Netherlands, https://thirona.eu). Percent emphysema (% emphysema) and percent air trapping (% air trapping) were defined as percentage of low attenuation lung areas below − 950 Hounsfield units on inspiratory CT and below − 856 Hounsfield units on expiratory CT, respectively [23–26]. Airway wall thickness was quantified as the square root of the wall area of a theoretical airway of 10 mm internal perimeter (Pi10) [27]. Vasculature measurements were performed on axial images of inspiratory CT from the COPDGene baseline study visit using DICOM software (OsiriX DICOM Viewer v4.0, www.osirix-viewer.com). Diameter of the main pulmonary artery was measured at its bifurcation, and diameter of the ascending aorta was measured at the same level in its maximum dimension. Pulmonary artery to aorta (PA/A) ratio was calculated, and relative PA enlargement was defined as a PA/A ratio greater than 1 [28, 29].

### Oxyhemoglobin saturation measurement

Oxyhemoglobin saturation (SpO_2_) was measured by a pulse oximeter placed on a finger without nail polish. When a strong pulse was demonstrated on oximetry the SpO_2_ readings were monitored for at least one minute and a median value was recorded. Resting SpO_2_ was measured with participants in a seated position and resting SpO_2_ was recorded as 82% if SpO_2_ dropped to 82% or lower. Supplemental oxygen was discontinued for at least five minutes prior to testing for participants who used oxygen therapy at rest [30]. For participants who completed a six-minute walk test (6MWT), exercise SpO_2_ was measured immediately after completion of the 6MWT. A participant could continue using supplemental oxygen during the 6MWT if the participant routinely used oxygen therapy with exercise.

### Long-term oxygen therapy (LTOT)

Participants who reported the use of LTOT on a questionnaire were further asked to provide information on the situations in which they used LTOT (at rest, and/or during sleep, and/or during exercise) as well as the number of hours of supplemental oxygen use on a typical day.

### COPD comorbidities

Common COPD comorbidities including congestive heart failure, diabetes, chronic kidney disease, and obstructive sleep apnea were self-reported. For participants reporting physician-diagnosed obstructive sleep apnea (OSA), further information on use of the use of a continuous positive airway pressure (CPAP) device was obtained.

### Statistical analysis

Resting SpO_2_ was categorized as normal (> 95%), mild hypoxemia (93–95%), moderate hypoxemia (89–92%), and severe hypoxemia (≤ 88%). Exercise-induced desaturation (EID) was defined as an SpO_2_ drop ≥ 4% from resting SpO_2_ and to < 90% at the end of 6MWT [31–33]. LTOT use was classified into four categories: (1) continuously (self-reported supplemental oxygen use both at rest and during sleep, or oxygen use at rest with more than 20 h per day); (2) nocturnally (during sleep only, or during sleep and exercise); (3) intermittently (at rest and/or during exercise without use during sleep); and (4) no use.

We used the Wilcoxon rank-sum test for continuous variables and Fisher’s exact test for categorical variables to compare baseline characteristics in participants with and without polycythemia. We used logistic regression to examine the association between polycythemia and potential risk factors, which were determined a priori based on clinical plausibility. Polycythemia risk factors and potential confounding variables included age, sex, race, BMI, enrollment center, current smoking status, pack-years of cigarette smoking, lung function parameters (FEV_1_% predicted and DLCO % predicted), quantitative chest CT measurements (% emphysema, % air trapping, Pi10), relative PA enlargement (PA/A ratio > 1), resting SpO_2_, EID, use of LTOT, and COPD comorbidities. Among all COPDGene clinical centers, Denver, Colorado (5280 feet above sea level) is significantly higher in altitude compared to other clinical centers and therefore enrollment center adjustment was based on participants being enrolled in a Denver versus non-Denver clinical center to control for differences in altitude. Multicollinearity among predictive variables was investigated using the variance inflation factor. We first examined the association between polycythemia and each risk factor, adjusting for the core covariates including age, sex, race, BMI, enrollment center, current smoking status, pack-years of cigarette smoking, and LTOT use. We then conducted a multivariable logistic regression that included all polycythemia risk factors with a suggestive statistical significance (*p* < 0.1) when adjusting for the core covariates. For all regression analyses we used participants with complete data for the outcome, all predictor variables, and core covariates. To reduce bias in estimates of coefficients, we used Firth’s logistic regression analyses when encountering rare events and/or quasi-separation of data [34, 35].

Self-identified race can serve as a proxy for one’s genetic ancestry though may be correlated with socioeconomic status (SES). We performed a sensitivity analysis to investigate the potential contribution of SES to our observed association of polycythemia with self-reported race, by including education level and income with race in the multivariable model. In a separate sensitivity analysis, we excluded participants with anemia (hemoglobin ≤ 13 g/dL in males and ≤ 12 g/dL in females) since anemic participants may have excess comorbidities and unmeasured confounders compared to non-anemic participants [36]. We then conducted another sensitivity analysis, excluding participants enrolled in the Denver clinical center to assess for possible confounding by pathophysiology features enriched in or unique to Denver participants. Due to a decreased number of participants with polycythemia in the analyses excluding Denver participants, we restricted the logistic regression to adjustment for age, sex, race, and variables with statistical significance in the multivariable logistic model for all participants. Given that COPD severity and LTOT use could differ between current and former smokers, as a secondary analysis we examined polycythemia risk factors stratified by smoking status. Given LTOT was mostly prescribed to patients with hypoxemia, we performed a subgroup analysis in participants with resting desaturation (SpO2 ≤ 95%) and/or EID versus normal resting SpO2 (> 95%) without EID.

A two-sided *p* value < 0.05 was considered statistically significant for all tests. All analyses were conducted using R version 3.6.3 (R Foundation for Statistical Computing, Vienna, Austria).

## Results

### Sample characteristics

Among 6284 former or current smokers with 10 pack-years or greater of tobacco consumption from the COPDGene study 5-year follow-up, 1928 participants (1054 males and 874 females) with moderate to very severe COPD and available hemoglobin and hematocrit data were included in this study. Of the 1928 participants, 128 (6.6%) individuals including 97 (9.2%) males and 31 (3.5%) females met the 2016 WHO diagnostic criteria for polycythemia. Individuals with polycythemia were more often male, non-Hispanic white, and enrolled in the Denver clinical center compared to individuals without polycythemia (Table 1). Comparing individuals with and without polycythemia, a greater proportion of individuals with polycythemia had severe airflow limitation (GOLD 3); however, a smaller proportion of individuals with polycythemia had very severe airflow limitation (GOLD 4). Participants with GOLD 4 airflow limitation were more often on LTOT (71.2% vs. 39.3%) compared to those with GOLD 3 airflow limitation. In addition, individuals with polycythemia more often had moderate or severe resting hypoxemia and less often reported use of continuous supplemental oxygen compared to individuals without polycythemia.

**Table 1:** Baseline characteristics according to the presence of polycythemia

Characteristic	Polycythemia (N = 128)	No polycythemia (N = 1800)	*P* value
Age (years)	66.0 (12.4)	67.9 (12.5)	0.17
Sex, male	97 (75.8)	957 (53.2)	**< 0.0001**
Race, non-Hispanic white	108 (84.4)	1355 (75.3)	**0.019**
Body mass index (kg/m^2^)	27.7 (6.9)	27.6 (7.9)	0.86
Pack-years of cigarette smoking	48.1 (24.0)	46.4 (30.0)	0.24
Enrollment center, Denver	43 (33.6)	268 (14.9)	**< 0.0001**
Hemoglobin (g/dL)	16.8 (0.9)	13.9 (1.9)	**< 0.0001**
Hematocrit (%)	50.3 (2.5)	42.0 (5.2)	**< 0.0001**
FEV_1_ percent predicted (%)	50.9 (27.5)	53.7 (29.0)	0.79
GOLD airflow limitation severity			**0.0051**
2	67 (52.3)	1033 (57.4)	
3	53 (41.4)	532 (29.6)	
4	8 (6.2)	235 (13.1)	
DLCO percent predicted (%) ^&^	61.2 (30.1)	61.7 (30.7)	0.86
Percent emphysema on CT (%) ^&^	6.0 (17.2)	7.3 (16.8)	0.87
Percent air trapping on CT (%) ^&^	33.6 (31.3)	35.8 (34.0)	0.39
Pi10 on CT (mm)	2.63 (0.83)	2.60 (0.72)	0.86
PA/A ratio, > 1.0 ^&^	13 (13.1)	285 (20.6)	0.090
Resting SpO_2_			**0.0042**
Normal	50 (39.1)	946 (52.6)	
Mild hypoxemia	43 (33.6)	558 (31.0)	
Moderate hypoxemia	22 (17.2)	201 (11.2)	
Severe hypoxemia	13 (10.2)	95 (5.3)	
Exercise-Induced desaturation ^&^	36 (29.3)	400 (23.2)	0.12
LTOT use			**0.012**
No use	101 (78.9)	1265 (70.3)	
Intermittent	3 (2.3)	46 (2.6)	
Nocturnal	16 (12.5)	196 (10.9)	
Continuous	8 (6.2)	293 (16.3)	
Obstructive sleep apnea/CPAP ^&^	9 (7.0)/7 (5.5)	213 (11.8)/108 (6.0)	0.11/0.17
Chronic kidney disease	0 (0)	65 (3.6)	**0.020**
Congestive heart failure	6 (4.7)	118 (6.6)	0.57
Diabetes mellitus	17 (13.3)	333 (18.5)	0.15

Continuous variables were presented as median (interquartile range); categorical variables were presented as number (percentage)

Bold numbers indicate values with statistical significance *P* < 0.05

*FEV*_*1*_ forced expiratory volume in 1 s, *GOLD* Global Initiative for Chronic Obstructive Lung Disease, *DLCO* diffusing capacity of lung for carbon monoxide, *Pi10* square root wall area of a theoretical airway of 10 mm internal perimeter, *PA/A ratio* ratio of the diameter of the pulmonary artery to the diameter of the aorta, *SpO*_2_ oxyhemoglobin saturation measured by oximetry, *LTOT* long-term oxygen therapy, *CPAP* continuous positive airway pressure

^&^ These variables had missing data

### Risk factors for secondary polycythemia

The association analyses of secondary polycythemia with each risk factor are shown in Table 2. After adjusting for core covariates, FEV1% predicted (odds ratio [OR], 0.98; 95% confidence interval [CI], 0.97–1.00) and DLCO % predicted (OR, 0.98; 95% CI, 0.96–0.99) were inversely associated with risk for polycythemia. Moderate (OR, 1.90; 95% CI, 1.02–3.53) and severe (OR, 3.32; 95% CI, 1.50–7.33) resting hypoxemia were associated with a higher risk for polycythemia. A trend toward association of quantitative CT % emphysema with polycythemia (OR, 1.02; *p* = 0.051) was found.

**Table 2:** Logistic regressions of each predictive variable on polycythemia

Predictive variable	OR	95% CI	*P* value
FEV_1_ percent predicted	0.98	0.97–1.00	**0.014**
DLCO percent predicted	0.98	0.96–0.99	**< 0.0001**
Percent emphysema on CT	1.02	1.00–1.04	0.051
Percent air trapping on CT	1.01	0.99–1.02	0.35
Pi10 on CT	0.99	0.70–1.41	0.96
PA/A ratio, > 1.0	0.80	0.42–1.52	0.50
Resting SpO_2_			
Normal (reference)	1.00		
Mild hypoxemia	1.30	0.83–2.04	0.24
Moderate hypoxemia	1.90	1.02–3.53	**0.044**
Severe hypoxemia	3.32	1.50–7.33	**0.0030**
Exercise-induced desaturation	1.48	0.95–2.31	0.081
Obstructive sleep apnea			
No (reference)	1.00		
Without CPAP use	0.25	0.06–1.06	0.060
With CPAP use	0.80	0.34–1.87	0.61
Congestive heart failure	1.00	0.42–2.40	1.00
Chronic kidney disease	0.08	0.00–1.31	0.076
Diabetes mellitus	0.68	0.39–1.19	0.18

The reported OR and *P* values are from logistic regression models for the association of secondary polycythemia with each predictive variable adjusted for a core set of covariates and confounders including age, sex, race, body mass index, enrollment clinical center, smoking status, pack-years of cigarette smoking, and use of long-term oxygen therapy

Bold numbers indicate values with statistical significance *P* < 0.05

*OR* odds ratio, *CI* confidence interval, *FEV*_*1*_ forced expiratory volume in 1 s, *DLCO* diffusing capacity of lung for carbon monoxide, *Pi10* square root wall area of a theoretical airway of 10 mm internal perimeter, *PA/A ratio* ratio of the diameter of the pulmonary artery to the diameter of the aorta, *SpO*_2_ oxyhemoglobin saturation measured by oximetry

The multivariable logistic regression for the association of polycythemia with all suggestive significant predictive variables (*p* < 0.1 in Table 2) and core covariates is shown in Table 3. In multivariable regression, polycythemia was associated with male sex, non-Hispanic white race (compared to African Americans), current smoking, and enrollment in the Denver clinical center. In addition, severe resting hypoxemia (OR, 3.50; 95% CI, 1.41–8.66) and impaired DLCO (OR, 1.28 for each 10-percent decrease in DLCO % predicted; 95% CI, 1.09–1.49) were associated with increased risk for polycythemia. Use of continuous (OR, 0.13; 95% CI, 0.05–0.35) or nocturnal (OR, 0.46; 95% CI 0.21–0.97) LTOT were associated with decreased risk for polycythemia.

**Table 3:** Multivariable logistic regression for polycythemia in COPD

Predictive variable	OR	95% CI	*P* value
Sex, male	3.60	2.20–5.90	**< 0.0001**
Race, non-Hispanic white	3.33	1.71–6.50	**0.0004**
Age at visit (year)	0.99	0.95–1.02	0.35
Center, Denver	4.42	2.38–8.21	**< 0.0001**
Body mass index (kg/m^2^)	1.03	0.99–1.07	0.19
Smoking status, current	2.55	1.49–4.38	**0.0007**
Pack-years of smoking	0.99	0.98–1.00	0.20
FEV_1_% predicted	1.00	0.98–1.01	0.59
DLCO % predicted	0.98	0.96–0.99	**0.0022**
Percent emphysema on CT	0.99	0.96–1.01	0.31
Resting SpO_2_			
Normal (reference)	1.00		
Mild desaturation	1.02	0.60–1.72	0.95
Moderate desaturation	1.43	0.68–2.99	0.35
Severe desaturation	3.50	1.41–8.66	**0.0068**
Exercise-induced desaturation	1.64	0.96–2.77	0.068
LTOT use			
No use (reference)	1.00		
Intermittent	0.49	0.12–2.04	0.32
Nocturnal	0.46	0.21–0.97	**0.041**
Continuous	0.13	0.05–0.35	**< 0.0001**
Obstructive sleep apnea			
No diagnosis (reference)	1.00		
Without CPAP treatment	0.39	0.11–1.41	0.15
With CPAP treatment	0.91	0.36–2.27	0.84
Chronic kidney disease	0.13	0.01–2.14	0.16

The reported OR and P values are from a single multivariable regression model

Bold numbers indicate values with statistical significance *P* < 0.05

*OR* odds ratio, *CI* confidence interval, *FEV1 * forced expiratory volume in 1 s, *DLCO* diffusing capacity of lung for carbon monoxide, *SpO2* oxyhemoglobin saturation measured by oximetry, *LTOT* long-term oxygen therapy, *CPAP* continuous positive airway pressure

After further adjustment of our multivariable model for education and income, race remained significantly associated with polycythemia with a similar effect size; however, neither education nor income was independently associated with polycythemia (Additional file 1: Table S1). The multivariable logistic regression results did not change significantly after excluding participants with anemia (Additional file 1: Table S2) or after excluding participants enrolled in the Denver clinical center (Additional file 1: Table S3).

### Stratified analyses by smoking status

The prevalence of polycythemia in former smokers (n = 1266) was 7.0% in males and 2.5% in females, and in current smokers (n = 662) was 13.6% in males and 5.5% in females. Compared with current smokers, formers smokers were older, more often non-Hispanic white, and more often enrolled in the Denver clinical center (Additional file 1: Table S4). Furthermore, former smokers appear to have more advanced COPD marked by more severe airflow limitation, lower DLCO % predicted, and more emphysema and air trapping on CT. In addition, compared with current smokers, former smokers more often had moderate or severe resting hypoxemia, and were more often on continuous or nocturnal LTOT.

**Fig. 1:**
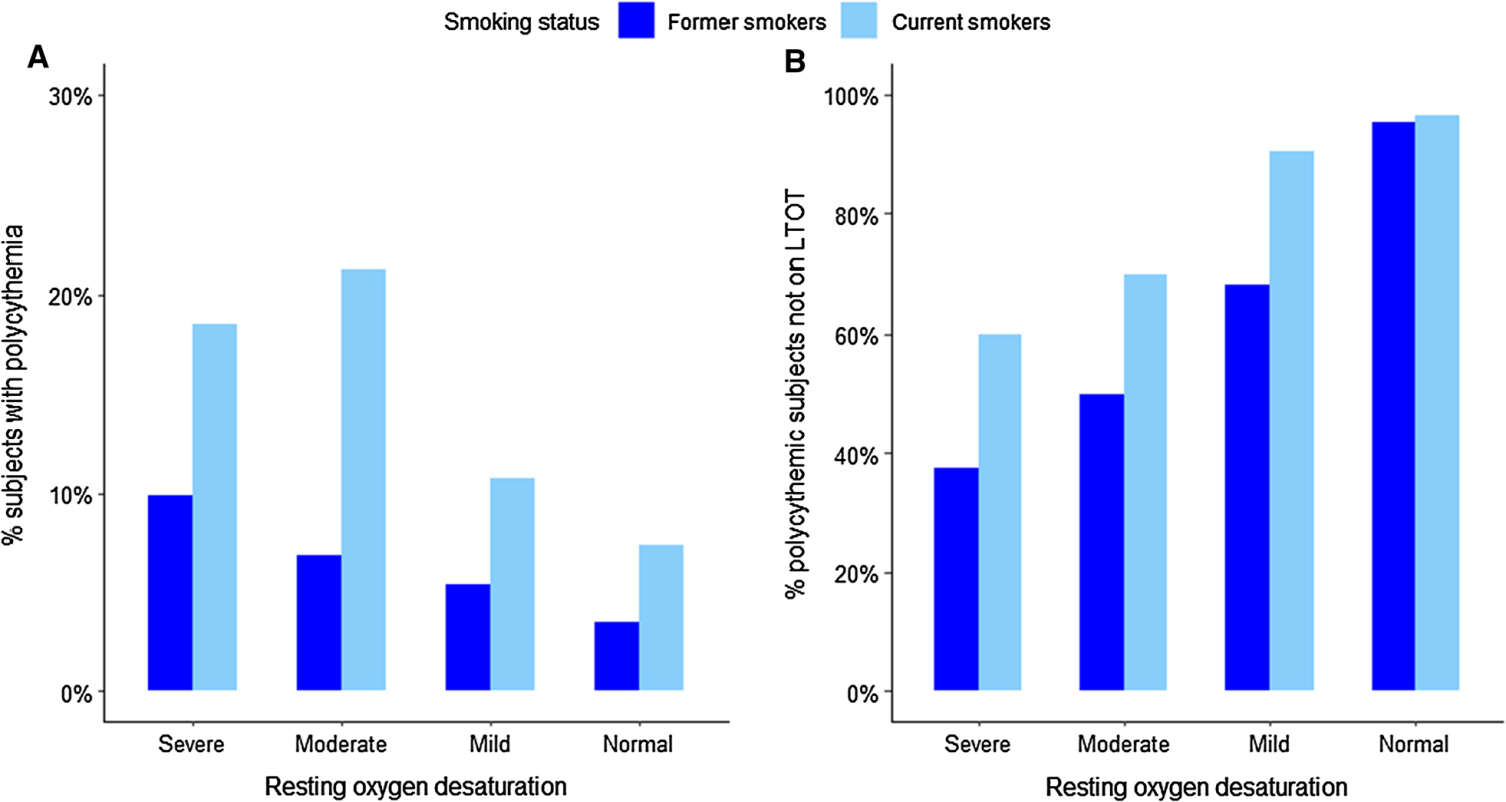
**A** Proportion of participants with polycythemia, **B** proportion of participants with polycythemia who were not on long-term oxygen therapy (LTOT) according to severity of resting oxygen desaturation stratified by smoking status

Among former smokers with severe resting hypoxemia, 9.9% had polycythemia and 37.5% (3/8) of them were not on LTOT; in contrast, 18.5% (5/27) of current smokers with severe resting hypoxemia had polycythemia and 60% (3/5) of them were not on supplemental oxygen (Fig. 1). For individuals with mild resting hypoxemia, 5.4% of former smokers and 10.7% of current smokers had polycythemia; and for individuals with moderate resting hypoxemia, 6.8% of former smokers and 21.3% of current smokers had polycythemia.

Multivariable logistic regression models were examined separately in former smokers and current smokers (Table 4). In former smokers, impaired DLCO (OR, 1.40 for each 10-percent decrease in DLCO % predicted; 95% CI, 1.13–1.72) was associated with increased risk for polycythemia, while using LTOT continuously (OR, 0.24; 95% CI, 0.08–0.71) was associated with decreased risk; there was not a significant association between polycythemia and any severity of resting hypoxemia. In current smokers, moderate resting hypoxemia (OR, 4.00; 95% CI, 1.28–12.54), severe resting hypoxemia (OR, 8.23; 95% CI, 1.93–35.07), and EID (OR, 2.66; 95% CI, 1.18–6.01) were associated with increased risk for polycythemia. Continuous LTOT use (OR, 0.02; 95% CI 0.00–0.39) was associated with decreased risk of polycythemia. DLCO was not significantly associated with polycythemia in current smokers.

**Table 4:** Multivariable logistic regression on polycythemia stratified by smoking status

Predictive variable	Former smokers (N = 1266)			Current smokers (N = 662)		
	OR	95% CI	*P* value	OR	95% CI	*P* value
Sex, male	4.60	2.29–9.25	** < 0.0001**	2.89	1.42–5.91	**0.0036**
Race, non-Hispanic white	2.96	1.02–8.59	**0.046**	3.60	1.56–8.30	**0.0026**
Age at visit (year)	0.98	0.94–1.02	0.34	0.99	0.95–1.04	0.81
Center, Denver	4.22	1.82–9.75	**0.0008**	5.59	2.16–14.45	**0.0004**
Body mass index (kg/m^2^)	1.01	0.95–1.07	0.80	1.05	0.99–1.12	0.079
Pack-years of smoking	0.99	0.98–1.00	0.21	1.00	0.98–1.01	0.71
FEV_1_% predicted	1.00	0.98–1.02	0.90	0.99	0.96–1.01	0.37
DLCO % predicted	0.97	0.95–0.99	**0.0016**	0.99	0.97–1.01	0.46
Percent emphysema on CT	0.97	0.94–1.01	0.17	1.01	0.96–1.06	0.81
Resting SpO_2_						
Normal (reference)	1.00			1.00		
Mild desaturation	0.93	0.45–1.91	0.84	0.98	0.47–2.07	0.96
Moderate desaturation	0.75	0.27–2.10	0.58	4.00	1.28–12.54	**0.017**
Severe desaturation	2.10	0.64–6.87	0.22	8.23	1.93–35.07	**0.0044**
Exercise-induced desaturation	1.19	0.60–2.39	0.62	2.66	1.18–6.01	**0.019**
LTOT use						
No use (reference)	1.00			1.00		
Intermittent	0.70	0.16–3.11	0.64	0.28	0.01–11.96	0.51
Nocturnal	0.63	0.26–1.53	0.31	0.47	0.12–1.83	0.28
Continuous	0.24	0.08–0.71	**0.0095**	0.02	0.00–0.39	**0.011**
Obstructive sleep apnea						
No diagnosis (reference)	1.00			1.00		
Without CPAP treatment	0.15	0.01–2.40	0.18	0.96	0.25–3.70	0.95
With CPAP treatment	1.24	0.39–3.95	0.71	0.39	0.08–1.82	0.23
Chronic kidney disease	0.20	0.01–3.29	0.26	0.38	0.02–6.50	0.50

The reported OR and P values are from a single multivariable regression model for each smoking status strata

Bold numbers indicate values with statistical significance *P* < 0.05

*OR* odds ratio, *CI* confidence interval, *FEV*_*1*_ forced expiratory volume in 1 s, *DLCO* diffusing capacity of lung for carbon monoxide, *SpO*_2_ oxyhemoglobin saturation measured by oximetry, *LTOT* long-term oxygen therapy, *CPAP* continuous positive airway pressure

### Stratified analyses by desaturation status

In subjects with desaturation at rest or exercise, nocturnal (OR, 0.44; 95% CI 0.19–0.99) and continuous LTOT (OR, 0.10; 95% CI 0.03–0.30) were associated with reduced risk for polycythemia (Additional file 1: Table S5). In participants without desaturation, we were not able to detect an association between LTOT use and polycythemia likely due to a very small number of participants (N = 2) who had polycythemia were on LTOT (Additional file 1: Table S6).

## Discussion

In this cohort of current and former smoking COPD patients from across the US, the overall prevalence of secondary polycythemia was 6.6% and was significantly higher in males, non-Hispanic whites, participants enrolled in Denver, and current smokers. A lower DLCO % predicted, severe resting hypoxemia, and exercise-induced desaturation (EID) were associated with a higher risk for polycythemia. Use of LTOT was associated with a lower risk for polycythemia.

Although secondary polycythemia has long been identified as a comorbidity in COPD, the prevalence of polycythemia in a contemporary sample of individuals with COPD was not well-established. Cote and colleagues reported a prevalence of polycythemia of 6% in 683 COPD outpatients from a single US clinical center [9]. Two other cohort studies, each less than 300 participants, evaluated the prevalence of polycythemia in European COPD outpatients [12, 13]. Studies based on hospitalized COPD patients or severe COPD patients receiving LTOT may have inaccurate polycythemia prevalence estimates due to selection and misclassification biases [10, 11, 37]. The COPDGene study prospectively enrolled a large number of non-Hispanic white and African American current and former smokers with and without COPD from multiple clinical centers across the US. COPDGene study participants covered a wide distribution of ages and had a balanced sex distribution.

Our study found a significantly higher risk of polycythemia in male COPD subjects compared to female subjects (even when accounting for COPD severity) an observation not well-documented previously. The underlying cause for the sex discrepancy in polycythemia prevalence is unknown. Of note, the cutoff values for hemoglobin and hematocrit in the updated 2016 WHO diagnostic criteria of polycythemia vera are less different by sex (0.5 g/dL difference in hemoglobin and 1% difference in hematocrit) than the former criteria (2 g/dL difference in hemoglobin). That said, even the current diagnostic criteria for polycythemia may not fully account for the baseline hemoglobin/hematocrit differences between males and females and may contribute to the observed sex difference in secondary polycythemia risk. Additionally, we found non-Hispanic whites with COPD were at higher risk for polycythemia compared with African Americans, which may be due to a lower baseline hemoglobin level in African Americans [38, 39]. Race remained significantly associated with polycythemia after adjusting for education and income. However, we are unable to rule out confounders including other more detailed socioeconomic measures and environmental effects. As self-reported race and genetic ancestry are highly correlated in the COPDGene study, it is possible that an ancestry specific effect on red cell traits may account for the association between self-reported race and polycythemia. Also, African Americans in the COPDGene study tended to have less severe COPD which might have further contributed to the observed racial difference in polycythemia risk, even with the adjustment for COPD severity in multivariable regression models [40].

Study participants enrolled in the Denver clinical center were found to have significantly higher risk for secondary polycythemia compared to those enrolled in clinical centers closer to sea level. Living at high altitude is associated with increased COPD mortality [41, 42], and healthy highlanders are more likely to have polycythemia [43], together suggesting a possible complex interplay between COPD, high altitude, and polycythemia. Denver participants had lower resting SpO_2_ compared to non-Denver participants after adjustment of COPD severity [44], which likely reflects high-altitude induced hypoxemia. In the present study, resting SpO_2_ and EID were adjusted for in regression analyses, though may not have sufficiently accounted for differences in the pattern and magnitude of hypoxemia experienced by Denver and non-Denver study participants. It is unclear whether high altitude is a risk factor for polycythemia independent from the effects of hypoxemia. In addition, Denver study participants tended to have more severe COPD, and may have high-altitude related environmental exposures, lung mechanics, and coexisting cardiopulmonary conditions different from non-Denver participants [45]. Those largely unknown factors may have mediated or confounded the association between Denver location and polycythemia despite the adjustment for lung function and quantitative CT measurements in our analyses. Though, in a sensitivity analysis of polycythemia associations excluding Denver participants, the remaining risk factors for polycythemia including male sex, non-Hispanic white race, and severe oxygen desaturation, were similar to the analysis that included Denver participants.

In the present study, severe resting hypoxemia was associated with higher risk for polycythemia whereas continuous or nocturnal use of LTOT were associated with lower risk for polycythemia, which indicates a strong association between uncorrected severe hypoxemia and polycythemia in COPD subjects. These findings are consistent with the longstanding understanding of chronic hypoxemia leading to secondary polycythemia in COPD. In a smoking-stratified analysis, former smokers were older and had more advanced COPD and hypoxemia severity compared to current smokers. Moderate resting hypoxemia, severe resting hypoxemia, and exercise-induced desaturation were associated with polycythemia in current smokers but not in former smokers. The proportion of participants with moderate or severe resting hypoxemia receiving LTOT was substantially lower in current smokers compared to former smokers, and continuous LTOT was associated with a decreased risk for polycythemia in both current and former smokers. Taken together, these findings suggest that differences in prescription of LTOT between current and former smokers (most likely due to safety issues in prescribing oxygen to current smokers) may account for the observed increased prevalence of secondary polycythemia in current smokers with COPD. Current smokers have higher and more variable COHb levels compared with non-smokers [46, 47]. SpO_2_ measured by oximetry may overestimate oxyhemoglobin level depending on the amount of COHb in blood [48]. Blood COHb level was not available in this study. As a result, the SpO_2_ measurement in our study may have upward bias in current smokers and result in a systematic less severe categorization of hypoxemia in current smokers.

The present study describes an independent association between impaired DLCO and increased risk for polycythemia, though the underlying mechanism accounting for this association remains unknown. Impaired DLCO can be related to more severe emphysema, concomitant interstitial lung disease, and pulmonary hypertension (PH) in COPD [49, 50]. In the current study, percent emphysema on CT showed a suggestive positive association with polycythemia in single variable regression but not in the multivariable regression which included DLCO. A possible explanation is that DLCO captured emphysema severity and additionally provided information of other underlying mechanisms contributing to polycythemia. Also, as a surrogate of invasively diagnosed PH, relative pulmonary artery (PA) enlargement on CT (PA/A ratio > 1) was not significantly associated with polycythemia in current study, though a direct measurement of pulmonary artery pressure was not available [29, 51]. First, it should be noted that the PA/A ratio measurement occurred approximately 5 years prior to the CBC measurements used to classify individuals as having secondary polycythemia, possibly introducing noise and misclassification bias to the association of PA/A ratio with polycythemia. Another possible explanation of the lack of association of PA/A ratio with polycythemia is that COPD patients with concomitant PH are prone to hospitalizations, which are associated with anemia [52, 53]. Notably, in stratified analysis DLCO was not significantly associated with polycythemia in current smokers. One possible explanation could be the association was mitigated by an increased variability of DLCO measurement in current smokers. COHb is known to affect DLCO measurement through occupation of hemoglobin binding sites and decreasing driving pressure of carbon monoxide transport from alveoli to blood, with a total effect of reducing measured DLCO [54]. In the present study DLCO was not corrected for COHb, which may have biased the estimation of association between DLCO and polycythemia in current smokers to an unknown direction or magnitude. Other possible causes of the null association between DLCO and polycythemia in current smokers include lack of power (same direction of effect estimate in current and former smokers), less severely impaired DLCO in current smokers compared with former smokers (smaller estimate of effect size in current smokers), and heavier cigarette smoking in study participants with less severe COPD.

Secondary polycythemia may have important clinical impact, including various comorbidities (e.g., pulmonary hypertension and venous thromboembolism) and mortality, on COPD patients. This study has important clinical implications related to the interpretation of and intervention on polycythemia in COPD patients. First, the present study clarifies that the prevalence of secondary polycythemia is considerable (~ 6%) in contemporary US COPD patients, especially in males and current smokers. Second, the presence of polycythemia in COPD patients may warrant an active screening of uncorrected severe hypoxemia, which is a predictor of mortality in COPD [55, 56]. LTOT use in COPD patients with severe resting hypoxemia improves survival and was also found to be associated with lower risk for polycythemia in our study [57–59]. Polycythemia can be easily discovered through a complete blood count, one of the most routinely acquired labs in clinical practice. As a result, a careful evaluation of polycythemia may provide a valuable opportunity to uncover uncorrected hypoxemia in COPD patients. Third, DLCO may be helpful in the evaluation of COPD patients with polycythemia, in whom a low DLCO might prompt an evaluation of concomitant interstitial lung disease or pulmonary vascular disease if hypoxemia alone fails to justify the presence of polycythemia.

The strengths of this study include a large cohort of contemporary COPD patients with extensively curated phenotypes including post-bronchodilator spirometry, DLCO, quantitative chest CT measurements, and LTOT use. CBCs were drawn from participants in stable clinical conditions rather than during COPD exacerbations or hospitalizations which limited misclassification of polycythemia based on acute medical events.

Limitations of this study include the cross-sectional design, which precludes inference of directionality or causality between exposure and outcome variables. Oxygen saturation was measured by pulse oximetry instead of arterial blood gas analysis, which might result in measurement bias [60]. Our available data did not allow us to definitely exclude polycythemia vera, though the prevalence of PV in US is quite low [61, 62]. Finally, LTOT use was self-reported, and information about the setting of initial prescription, follow-up adjustment, or barriers to LTOT use was not available.

## Conclusions

In conclusion, in a large sample of current and former smoking individuals with COPD and moderate to very severe airflow limitation, we found a substantial prevalence of polycythemia. The prevalence of polycythemia was significantly higher in males and current smokers. Severe resting hypoxemia, impaired DLCO, and living at high altitude (Denver) were risk factors for polycythemia; whereas, continuous or nocturnal LTOT use were protective against secondary polycythemia in COPD. The longitudinal clinical impact of polycythemia and whether additional risk assessment for polycythemia can lead to improved COPD outcomes and management, such as a more in-depth evaluation for chronic hypoxemia, requires further study.

## Supplementary Information

**Additional file 1**. **Table S1.** Multivariable logistic regression for polycythemia in COPD with further adjustment for education and income. **Table S2**. Multivariable logistic regression on polycythemia in subjects without anemia. **Table S3**. Multivariable logistic regression on polycythemia in subjects not enrolled in Denver. **Table S4**. Baseline characteristics by current smoking status. **Table S5**. Multivariable logistic regression on polycythemia stratified by desaturation status. **Table S6**. Distribution of Long-term oxygen therapy uses on polycythemia by desaturation status.

**Additional file 2**. Investigator information and full acknowledgements for the COPDGene study.

## Data Availability

The datasets used and/or analyzed during the current study are available from the corresponding author on reasonable request.

## Abbreviations

6MWT: Six-minute walk test
BMI: Body mass index
CBC: Complete blood count
CI: Confidence interval
COHb: Carboxyhemoglobin
COPD: Chronic obstructive pulmonary disease
COPDGene: Genetic Epidemiology of COPD
CPAP: Continuous positive airway pressure
CT: Computed tomography
DLCO: Diffusing capacity of lung for carbon monoxide
EID: Exercise-induced desaturation
FEV1: Forced expiratory volume in 1 s
FVC: Forced vital capacity
GOLD: Global Initiative for Chronic Obstructive Lung Disease
LTOT: Long-term oxygen therapy
OR: Odds ratio
OSA: Obstructive sleep apnea
PA: Pulmonary artery
PA/A ratio: Pulmonary artery to aorta diameter ratio
PH: Pulmonary hypertension
Pi10: Square root wall area of a theoretical airway of 10 mm internal perimeter
PV: Polycythemia vera
SES: Socioeconomic status
SpO2: Oxyhemoglobin saturation
WHO: World Health Organization
