# Supplementary material for: Secondary polycythemia in chronic obstructive pulmonary disease: prevalence and risk factors
Source: BMC Pulm Med. 2021 Jul 14;21:235. doi: 10.1186/s12890-021-01585-5 (PMC8278596; doi:10.1186/s12890-021-01585-5)
Supplement: Supplementary file 1 — Additional file 1. Table S1. Multivariable logistic regression for polycythemia in COPD with further adjustment for education and income. Table S2. Multivariable logistic regression on polycythemia in subjects without anemia. Table S3. Multivariable logistic regression on polycythemia in subjects not enrolled in Denver. Table S4. Baseline characteristics by current smoking status. Table S5. Multivariable logistic regression on polycythemia stratified by desaturation status. Table S6. Distribution of Long-term oxygen therapy uses on polycythemia by desaturation status. [file 12890_2021_1585_MOESM1_ESM.docx]

Additional file 1: Table S1. Multivariable logistic regression for polycythemia in COPD with further adjustment for education and income.

| Predictive variable | OR | 95% CI | *P* value |
| --- | --- | --- | --- |
| Sex, male | 4.45 | 2.56-7.73 | **<0.0001** |
| Race, non-Hispanic white | 3.19 | 1.51-6.74 | **0.0024** |
| Education |  |  |  |
| High school and below (reference) | 1.00 |  |  |
| College | 1.03 | 0.61-1.72 | 0.92 |
| Graduate school | 0.59 | 0.22-1.60 | 0.30 |
| Income (US dollar) |  |  |  |
| <15,000 (reference) | 1.00 |  |  |
| 15,000-35,000 | 1.28 | 0.68-2.41 | 0.44 |
| 35,000-50,000 | 1.28 | 0.60-2.74 | 0.52 |
| 50,000-75,000 | 1.64 | 0.72-3.74 | 0.24 |
| >75,000 | 0.92 | 0.36-2.38 | 0.87 |
| Age at visit (year) | 0.99 | 0.95-1.02 | 0.46 |
| Center, Denver | 5.09 | 2.64-9.81 | **<0.0001** |
| Body mass index (kg/m^2^) | 1.04 | 1.00-1.09 | 0.06 |
| Smoking status, current | 2.36 | 1.32-4.22 | **0.0037** |
| Pack-years of smoking | 0.99 | 0.98-1.00 | 0.26 |
| FEV_1_ % predicted | 1.00 | 0.98-1.02 | 0.93 |
| DLCO % predicted | 0.97 | 0.96-0.99 | **0.0014** |
| Percent emphysema on CT | 0.99 | 0.95-1.02 | 0.35 |
| Resting SpO_2_ |  |  |  |
| Normal (reference) | 1.00 |  |  |
| Mild desaturation | 1.14 | 0.65-2.01 | 0.64 |
| Moderate desaturation | 1.45 | 0.64-3.28 | 0.37 |
| Severe desaturation | 3.87 | 1.46-10.29 | **0.0067** |
| Exercise-induced desaturation | 1.59 | 0.89-2.82 | 0.12 |
| LTOT use |  |  |  |
| No use (reference) | 1.00 |  |  |
| Intermittent | 0.24 | 0.04-1.36 | 0.11 |
| Nocturnal | 0.37 | 0.16-0.82 | **0.015** |
| Continuous | 0.12 | 0.04-0.33 | **<0.0001** |
| Obstructive sleep apnea |  |  |  |
| No diagnosis (reference) | 1.00 |  |  |
| without CPAP treatment | 0.43 | 0.12-1.58 | 0.20 |
| with CPAP treatment | 0.81 | 0.31-2.08 | 0.66 |
| Chronic kidney disease | 0.13 | 0.01-2.16 | 0.16 |

The reported OR and P values are from a single multivariable regression model.

OR=odds ratio, CI=confidence interval, FEV_1_=forced expiratory volume in 1s, DLCO=diffusing capacity of lung for carbon monoxide, SpO2= oxyhemoglobin saturation measured by oximetry, LTOT=long-term oxygen therapy, CPAP=continuous positive airway pressure

Additional file 1: Table S2. Multivariable logistic regression on polycythemia in subjects without anemia (N=1635).

| Predictive variable | OR | 95% CI | *P* value |
| --- | --- | --- | --- |
| Sex, male | 3.81 | 2.32-6.28 | **<0.0001** |
| Race, non-Hispanic white | 3.07 | 1.55-6.06 | **0.0013** |
| Age at visit (year) | 0.99 | 0.96-1.02 | 0.42 |
| Center, Denver | 4.08 | 2.19-7.59 | **<0.0001** |
| Body mass index (kg/m^2^) | 1.03 | 0.98-1.07 | 0.22 |
| Smoking status, current | 2.45 | 1.41-4.24 | **0.0014** |
| Pack-years of smoking | 0.99 | 0.99-1.00 | 0.23 |
| FEV_1_ % predicted | 0.99 | 0.98-1.01 | 0.49 |
| DLCO % predicted | 0.98 | 0.96-0.99 | **0.0021** |
| Percent emphysema on CT | 0.98 | 0.95-1.01 | 0.24 |
| Resting SpO_2_ |  |  |  |
| Normal (reference) | 1.00 |  |  |
| Mild desaturation | 1.02 | 0.60-1.73 | 0.95 |
| Moderate desaturation | 1.39 | 0.66-2.91 | 0.39 |
| Severe desaturation | 3.93 | 1.54-10.02 | **0.0041** |
| Exercise-induced desaturation | 1.70 | 1.00-2.90 | 0.051 |
| LTOT use |  |  |  |
| No use (reference) | 1.00 |  |  |
| Intermittent | 0.46 | 0.11-2.00 | 0.30 |
| Nocturnal | 0.44 | 0.20-0.96 | **0.038** |
| Continuous | 0.15 | 0.06-0.42 | **0.0003** |
| Obstructive sleep apnea |  |  |  |
| No diagnosis (reference) | 1.00 |  |  |
| without CPAP treatment | 0.37 | 0.10-1.36 | 0.13 |
| with CPAP treatment | 0.88 | 0.35-2.24 | 0.80 |
| Chronic kidney disease | 0.18 | 0.01-2.91 | 0.23 |

OR=odds ratio, CI=confidence interval, FEV_1_=forced expiratory volume in 1s, DLCO=diffusing capacity of lung for carbon monoxide, SpO2= oxyhemoglobin saturation measured by oximetry, LTOT=long-term oxygen therapy, CPAP=continuous positive airway pressure

Additional file 1: Table S3. Multivariable logistic regression on polycythemia in subjects not enrolled in Denver (N=1617).

| Predictive variable | OR | 95% CI | *P* value |
| --- | --- | --- | --- |
| Sex, male | 2.87 | 1.65-4.98 | **0.0002** |
| Race, non-Hispanic white | 3.64 | 1.74-7.59 | **0.0006** |
| Age at visit (year) | 0.98 | 0.95-1.02 | 0.27 |
| Smoking status, current | 2.05 | 1.20-3.50 | **0.0089** |
| DLCO % predicted | 0.98 | 0.97-0.99 | **0.0032** |
| Resting SpO_2_ |  |  |  |
| Normal (reference) | 1.00 |  |  |
| Mild desaturation | 0.99 | 0.57-1.70 | 0.97 |
| Moderate desaturation | 1.66 | 0.68-4.06 | 0.27 |
| Severe desaturation | 3.76 | 1.02-13.86 | **0.046** |
| LTOT use |  |  |  |
| No use (reference) | 1.00 |  |  |
| Intermittent | 0.82 | 0.20-3.34 | 0.78 |
| Nocturnal | 0.60 | 0.22-1.64 | 0.32 |
| Continuous | 0.26 | 0.07-0.97 | **0.045** |

OR=odds ratio, CI=confidence interval, DLCO=diffusing capacity of lung for carbon monoxide, SpO2= oxyhemoglobin saturation measured by oximetry, LTOT=long-term oxygen therapy

Additional file 1: Table S4. Baseline characteristics by current smoking status.

| Characteristic | Former smokers (N=1266) | Current smokers (N=662) | *P* value |
| --- | --- | --- | --- |
| Polycythemia | 63 (5.0) | 65 (9.8) | **0.0001** |
| Age (years) | 70.8 (10.7) | 62.5 (10.3) | **<0.0001** |
| Sex, male | 701 (55.4) | 353 (53.3) | 0.41 |
| Race, non-Hispanic white | 1049 (82.9) | 414 (62.5) | **<0.0001** |
| Body mass index (kg/m^2^) | 28.1 (7.8) | 26.0 (8.4) | **<0.0001** |
| Pack-years of cigarette smoking | 46.3 (32.0) | 46.7 (24.5) | 0.40 |
| Enrollment center, Denver | 246 (19.4) | 65 (9.8) | **<0.0001** |
| FEV_1_ percent predicted (%) | 50.6 (29.3) | 59.0 (24.6) | **<0.0001** |
| GOLD airflow limitation severity |  |  | **<0.0001** |
| 2 | 655 (51.7) | 445 (67.2) |  |
| 3 | 412 (32.5) | 173 (26.1) |  |
| 4 | 199 (15.7) | 44 (6.6) |  |
| DLCO percent predicted (%) ^&^ | 60.2 (32.9) | 63.3 (26.9) | **0.0026** |
| Percent emphysema on CT (%) ^&^ | 10.5 (19.7) | 3.4 (9.1) | **<0.0001** |
| Percent gas trap on CT (%) ^&^ | 40.1 (32.9) | 27.8 (28.8) | **<0.0001** |
| Pi10 on CT (mm) | 2.55 (0.64) | 2.70 (0.86) | **<0.0001** |
| PA/A ratio, >1.0 ^&^ | 214 (16.9) | 84 (12.7) | 0.44 |
| Resting SpO_2_ |  |  | **<0.0001** |
| Normal | 604 (47.7) | 392 (59.2) |  |
| Mild hypoxemia | 405 (32.0) | 196 (29.6) |  |
| Moderate hypoxemia | 176 (13.9) | 47 (7.1) |  |
| Severe hypoxemia | 81 (6.4) | 27 (4.1) |  |
| Exercise-induced desaturation ^&^ | 334 (27.5) | 102 (16.0) | **<0.0001** |
| LTOT use |  |  | **<0.0001** |
| No use | 788 (62.2) | 578 (87.3) |  |
| Intermittent | 42 (3.3) | 7 (1.1) |  |
| Nocturnal | 170 (13.4) | 42 (6.3) |  |
| Continuous | 266 (21.0) | 35 (5.3) |  |

Continuous variables were presented as median (interquartile range); categorical variables were presented as number (percentage). FEV_1_= forced expiratory volume in 1s, GOLD= Global Initiative for Chronic Obstructive Lung Disease, DLCO= diffusing capacity of lung for carbon monoxide, Pi10= square root wall area of a theoretical airway of 10 mm internal perimeter, PA/A ratio= ratio of the diameter of the pulmonary artery to the diameter of the aorta, SpO_2_= oxyhemoglobin saturation measured by oximetry, LTOT= long-term oxygen therapy.

& These variables had missing data.

Additional file 1: Table S5. Multivariable logistic regression on polycythemia stratified by desaturation status.

| Predictive variable | Desaturation (N=1078) | | | No desaturation (N=850) | | |
| --- | --- | --- | --- | --- | --- | --- |
|  | OR | 95% CI | *P* value | OR | 95% CI | *P* value |
| Sex, male | 2.57 | 1.42-4.65 | **0.0018** | 6.43 | 2.39-17.30 | **0.0002** |
| Race, non-Hispanic white | 3.12 | 1.27-7.68 | **0.013** | 3.91 | 1.36-11.28 | **0.012** |
| Age at visit (year) | 0.99 | 0.95-1.03 | 0.61 | 0.99 | 0.94-1.04 | 0.63 |
| Center, Denver | 6.67 | 3.47-12.84 | **<0.0001** | 6.26 | 1.69-23.14 | **0.0060** |
| Body mass index (kg/m^2^) | 1.04 | 0.99-1.10 | 0.11 | 1.01 | 0.95-1.08 | 0.67 |
| Smoking status, current | 2.77 | 1.38-5.55 | **0.0042** | 2.26 | 0.95-5.36 | 0.064 |
| Pack-years of smoking | 1.00 | 0.98-1.01 | 0.39 | 0.99 | 0.97-1.01 | 0.18 |
| FEV_1_ % predicted | 0.99 | 0.97-1.01 | 0.47 | 1.00 | 0.97-1.03 | 1.00 |
| DLCO % predicted | 0.96 | 0.94-0.98 | **0.0006** | 0.98 | 0.96-1.01 | 0.14 |
| Percent emphysema on CT | 0.98 | 0.95-1.02 | 0.35 | 0.99 | 0.93-1.04 | 0.66 |
| LTOT use * |  |  |  |  |  |  |
| No use (reference) | 1.00 |  |  | 1.00 |  |  |
| Intermittent | 0.50 | 0.11-2.40 | 0.39 | 0.40 | 0.07-2.25 | 0.30 |
| Nocturnal | 0.44 | 0.19-0.99 | **0.046** |  |  |  |
| Continuous | 0.10 | 0.03-0.30 | **<0.0001** |  |  |  |

Desaturation = resting SpO2 ≤95% or exercise induced desaturation.

The reported OR and P values are from a single multivariable regression model for each smoking status strata.

OR=odds ratio, CI=confidence interval, FEV_1_=forced expiratory volume in 1s, DLCO=diffusing capacity of lung for carbon monoxide, SpO2= oxyhemoglobin saturation measured by oximetry, LTOT=long-term oxygen therapy

* For participants without desaturation, LTOT use was categorized into no use (refence) versus any use (intermittent, nocturnal, or continuous use) due to only 2 participants with polycythemia were on LTOT.

Additional file 1: Table S6. Distribution of Long-term oxygen therapy uses on polycythemia by desaturation status.

|  | Desaturation (N=1078) | | | No desaturation (N=850) | | |
| --- | --- | --- | --- | --- | --- | --- |
|  | Polycythemia  (N=87) | No polycythemia  (N=991) | *P* value | Polycythemia  (N=41) | No polycythemia  (N=809) | *P* value |
| LTOT use |  |  | **0.0053** |  |  | 0.45 |
| No use | 62 | 586 |  | 39 | 679 |  |
| Intermittent | 3 | 32 |  | 0 | 14 |  |
| Nocturnal | 15 | 145 |  | 1 | 51 |  |
| Continuous | 7 | 226 |  | 1 | 65 |  |

Desaturation = resting SpO2 ≤95% or exercise induced desaturation.

LTOT=long-term oxygen therapy
